# 姜黄素通过下调miR-186^*^促进人肺腺癌细胞A549/DDP凋亡

**DOI:** 10.3779/j.issn.1009-3419.2010.04.06

**Published:** 2010-04-20

**Authors:** 妮 唐, 艰 张, 永平 杜

**Affiliations:** 1 710032 西安，第四军医大学第一附属医院中医科 Department of Traditional Medicine, the First Hospital Affiliated to Fourth Military Medical University, Xi'an 710032, China; 2 710032 西安，第四军医大学第一附属医院呼吸科 Department of Respiratory Medicine, the First Hospital Affiliated to Fourth Military Medical University, Xi'an 710032, China

**Keywords:** 姜黄素, miR-186^*^, A549/DDP, 凋亡, Curcumin, miR-186^*^, A549/DDP, Apoptosis

## Abstract

**背景与目的:**

姜黄素是从姜黄科植物的根茎中提取的一种天然化合物。体内外的临床前研究表明，其具有抗炎、抗氧化、抗肿瘤等多种作用。miR-186^*^是通过microarray芯片技术发现的在人肺腺癌细胞A549/DDP细胞中高表达的基因。本研究旨在阐明姜黄素是否可通过调控miR-186^*^的表达而促进A549/DDP细胞的凋亡。

**方法:**

microarray芯片技术检测经姜黄素、DMSO分别处理后A549/DDP细胞中microRNAs（miRNAs）表达量的变化；实时定量PCR验证从microarray芯片中筛选出来的明显差异表达的miRNAs；最后，流式细胞仪法检测明显差异表达的miRNAs调控的细胞凋亡，MTT法检测细胞存活率。

**结果:**

microarray芯片技术结果显示：与DMSO对照组比较，姜黄素处理后miR-186^*^的表达明显下调。实时定量PCR验证了microarray芯片技术检测的结果。流式细胞仪法检测miR-186^*^调控的细胞凋亡，MTT法检测细胞存活率：姜黄素处理后，与对照组比较，miR-186^*^的表达明显下调，从而加速A549/DDP细胞的凋亡，细胞存活率下降；转染miR-186^*^的mimetics和其对照后，与对照组比较，miR-186^*^的表达上调，从而减少A549/DDP细胞的凋亡，细胞存活率增加。

**结论:**

姜黄素调控miRNAs的表达可能是其促肺癌细胞凋亡的重要机制之一。

## Abstract (en)

**Background and objective:**

Curcumin, a natural compound, is derived from the rthizom of Curcuma longa. *In vitro* and *in vivo* preclinical studies have shown its anti-inflammatory, antioxidant, anticancer activities and so on. miR-186^*^, which was found by microarray technology, was highly expressed in lung carcinoma cells A549/DDP. The aim of this study is to illustrate whether Curcumin could promote the apoptosis of A549/DDP cells through regulating the expression of miR-186^*^.

**Methods:**

An oligonucleotide microarray chip was used to profile microRNA (miRNA) expressions in A549/DDP cells treated with and without Curcumin. The significantly differentially expressed miRNA, which was selected from microarray chip, validated by quantitative real-time PCR. Ultimately, the remarkably expressed miRNA modulated the apoptosis assaying by flow cytometry expriments and the survival rate was measured by MTT method.

**Results:**

The microarray chip results demonstrated: Curcumin altered the expression level of miRNAs compared with untreated control in A549/DDP cell line, miR-186^*^ was significantly down-regulated after Curcumin treatment, which confirmed by quantitative real-time PCR. Downregulation of miR-186^*^ expression by curcumin elevated the apoptosis, and the survival rate of A549/DDP cells decreased; but up-regulation of miR-186^*^ expression by transfection its mimics restrained the apoptosis, the survival rate of A549/DDP cells increased, which were assayed by flow cytometry expriments and MTT method.

**Conclusion:**

Modulation of miRNAs expression may be an important mechanism underlying the biological roles of Curcumin.

据报道，吸烟导致肺癌的发病率逐渐增多，然而随着铂类作为一线抗癌药物不断应用于临床，其耐药已成为目前关注的热点。发现一种既安全又有效的抗癌药物并使其应用于临床显得非常重要。姜黄素是从姜黄科植物的根茎中提取的一种酚性化合物^[1, 2]^，其各种药理作用已有报道，包括抗炎、抗氧化及抗肿瘤等^[3-5]^。

microRNAs（miRNAs）是一组生物体基因组编码的内源性非编码小分子RNA，成熟的miRNA长度为19 nt-25 nt。miRNAs作为最初始的转录本经过RNase Ⅲ Drosha和Dicer序列加工而被转录^[6]^。每个miRNA可调控许多mRNA，但是在机体组织中，miRNA的所有功能均为被系统地阐述^[7]^，研究^[8]^发现，在肿瘤的发展中，异常表达的miRNA起着非常重要的作用。有关肿瘤发展过程中基因变化的详细知识或许对早期临床诊断和治疗措施的构建有很大帮助。miR-186^*^是一个在人肺腺癌耐药细胞中新发现的基因，它在肿瘤细胞中的作用机制尚未见报道。本研究旨在阐明姜黄素是否可通过下调mir-186^*^的表达而抑制A549/DDP细胞生长的生物学功能。

## 1 材料与方法

### 1.1 细胞系与培养

人肺腺癌细胞A549/DDP取自第三军医大学呼吸病研究所实验室，细胞用RPMI-1640培养液加10%的胎牛血清和100 U/mL青霉素或100 µg/mL链霉素于37 ℃、5%CO_2_的孵箱中孵育。为了保持A549/DDP细胞耐药的表型，每次换液时在细胞培养液中均添加顺铂（终浓度为1 μg/mL）。细胞用胰酶消化后计数。

### 1.2 miRNA芯片检测

miRNA标记及miRNA点阵杂交实验开始前，将不加顺铂的A549/DDP细胞培养1周后，消化并记数。总RNA的提取按照Invitrogen公司的RNA提取试剂盒说明进行。miRNA的进一步纯化按照Ambion公司的mirVana^TM^ miRNA纯化试剂盒进行。自A549/DDP细胞系中提取的RNA用Hy3标记。方法：按照Exiqon公司的miRCURY^TM^ Array power标记试剂盒进行。RNA各自在miRCUPY^TM^ LNA芯片上的杂交按照说明书进行（v 8.0, Exiqon）。微点阵图像的扫描要求用GenePix 4000B扫描，数据的分析用GenePix Pro 6.0软件及Excel表完成。以上实验重复4次。

### 1.3 实时定量PCR分析从microarray芯片中筛选出的明显表达的miRNA

RNA的Trizol法提取用Invitrogen公司的RNA提取试剂盒进行，每个样品重复3次，包括减去反转录的内参（U6），以便评估基因组DNA。用于实时定量PCR反应体系的试剂包括：2.0 µL 10×RT缓冲液（Ambion）、2.0 µL dNTPs、2.5 mM each（Ambion）、1 µL 5×RT引物（如表 1所示）、0.3 µL RNase抑制蛋白40 U/µL（Ambion）、2 µL wt-MMLV-RT、100 U/µL（Ambion），最终10 µL的液体里含有500 pg总RNA。实时定量PCR中总RNAs的提取与microarray分析中总RNAs来自同种样品，然后，显著表达的miRNAs和内参U6用ABI Prism 7600探测系统逆转录和扩增。样品置于96孔板中，95 ℃、5 min，95 ℃、10 s，60 ℃、20 s，72 ℃、20 s，分别进行40个循环。显著表达的miRNA的定量数据分析采用2^-△△Ct^^[9]^法进行分析（表 1）。

**1:** miR-186^*^和U6引物序列 The primers of miR-186^*^ and U6

Primer	Sequence
U6 (Forward primer)	5'GCTTCGGCAGCACATATACTAAAAT3'
U6 (Reverse primer)	5'CGCTTCACGAATTTGCGTGTCAT3'
miR-186^*^ (Forward primer)	5'CCCGATAAAGCTAGATAACC3'
miR-186^*^ (Reverse piimer)	5'CAGTGCGTGTCGTGGAGT3’

### 1.4 miRNA转染

转染试剂（DharmaFECT1）购买自Dharmacon。mir-186^*^ mimetics及mimetic controls（均为冻干寡聚核苷酸粉形式）由Dharmacon公司合成。miRNA mimetics是小的单股的miRNA寡聚核苷酸，模仿内源性的成熟miRNA。有活性的位点进入miRNA的作用途径调控目标miRNA的表达。转染效率的简单估计由转染入细胞Dharamcon公司的双股siRNA荧光素酶，但未做功能方面的分析。

#### 1.4.1 细胞培养

用于实验的细胞消化后应被计数，在无抗生素的培养液中培养细胞。96孔板中，每孔培养液的体积是100 μL，细胞密度均匀适合，然后放置于37 ℃、5% CO_2_的孵箱中过夜。

#### 1.4.2 转染

将miRNA mimetics和mimetic controls（5 nmol）分别溶解在RNase-free蒸馏水中。在1号管中溶解2 μM小干扰RNA，在2号管中加入转染试剂，然后分别在以上两管中加入无血清的培养液。再将两管中的溶液上下颠倒混匀后，室温放置5 min。最后将1号管中的液体吸出加到2号管中混匀后，再加入足够的无抗生素的培养液，室温放置20 min。之后，将原来的96孔板中的培养液弃掉，再将混合后的溶液以每孔100 μL的体积加到96孔板中。转染后的细胞放置于37 ℃、5% CO_2_的孵箱中孵育48 h。

### 1.5 凋亡的检测

#### 1.5.1 流式细胞仪法检测细胞凋亡

凋亡的量用PI和Annexin V-FITC的双重染色法测定。PI和Annexin V-FITC试剂盒购自B.D公司。姜黄素处理与转染的细胞及其各自的对照组细胞用预冷PBS洗涤2次，再用5 μL Annexin V-FITC和10 μL PI室温孵育30 min，然后细胞用BectonDickinson FACS-420流式细胞仪分析。其发射的刺激波长分别是488 nm和525 nm。以上实验重复3次。

#### 1.5.2 MTT法检测细胞存活率

15 μmol/L姜黄素处理和用0.02%DMSO作为对照处理A549/DDP细胞，24 h后，用预冷PBS洗涤2次后，分别依次加入MTT溶液20 μL，4 h后，吸取上清液，每孔加入150 mL DMSO，选择490 nm波长，在酶联免疫检测仪上测定各孔的吸光度。转染miR-186^*^ mimetics和mimetic controls，48 h后，用预冷PBS洗涤2次后，分别依次加入MTT溶液20 μL，4 h后，吸取上清液，每孔加入150 mL DMSO，选择490 nm波长，在酶联免疫检测仪上测定各孔的吸光度。以上实验分别重复4次，存活率（survival rate）=处理组OD/对照组OD。

### 1.6 统计学方法

采用SPSS 11.0统计软件进行处理，以MEAN±SD表示，组间比较采用*q*检验，*P* < 0.05为差异有统计学意义。

## 2 结果

### 2.1 姜黄素处理后miRNAs的表达水平变化

microarray实验共获两张芯片，每个miRNAs的结果在芯片上重复4次，芯片的结果全做标准化处理。筛选差异表达较明显的miRNAs：15 μmol/L姜黄素处理组与0.02%DMSO对照组比较后，上调或下调30%的miRNAs并且这些miRNAs的表达量在A549/DDP细胞中均超过0.2的才被选择。其中6个miRNAs的表达显著下调，6个miRNAs的表达显著上调（*P* < 0.05）。在这些miRNAs中，mir-30b显著上调56.5%，miR-186^*^显著下调45.2%（图 1）。

**1:**
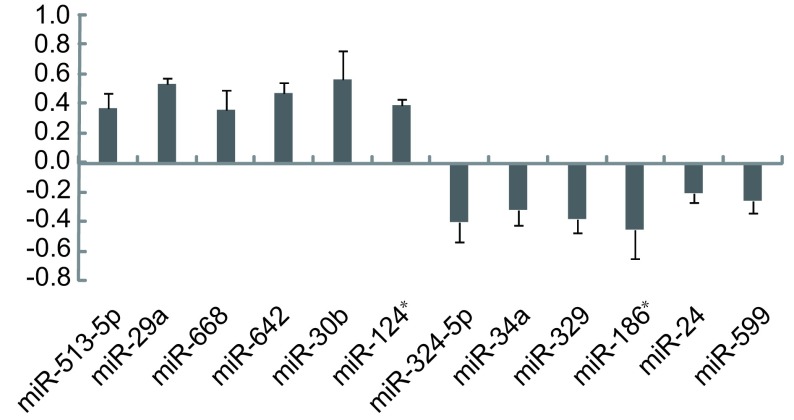
与DMSO对照组相比，姜黄素处理后差异表达明显的miRNAs The significantly differentially expressed miRNAs after Curcumin treatment compared to DMSO controls

### 2.2 实时定量PCR验证miR-186^*^的表达

microarray分析结果：与DMSO对照组相比，姜黄素处理后，miR-186^*^表达下调45.2%（*n*=3, *P* < 0.05）。实时定量PCR结果：姜黄素处理后，miR-186^*^的表达量为0.7±0.1，未处理组miR-186^*^的表达量为1.4±0.2。与DMSO对照组相比，姜黄素处理后，miR-186^*^的表达下调70.0%（*n*=3, *P* < 0.05）。实时定量PCR的实验结果进一步验证了microarray分析结果（图 2）。

**2:**
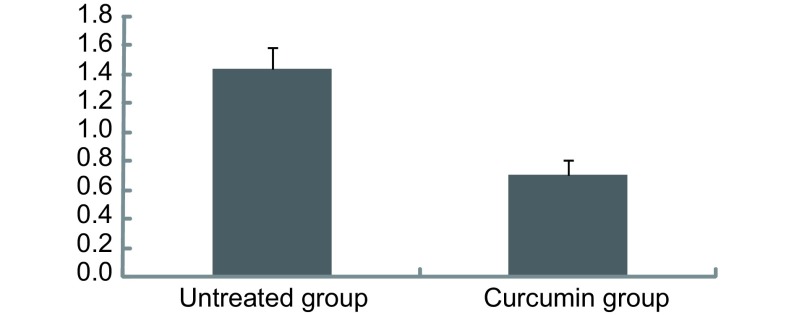
实时定量PCR检测miR-186^*^表达 The profile of miR-186^*^ assayed by quantitative real-time PCR

### 2.3 流式细胞仪检测由miR-186^*^调控的细胞凋亡

15 μmol/L姜黄素处理和用0.02%DMSO作为对照处理A549/DDP细胞，孵育24 h后收集细胞；转染miR-186^*^ mimetics和mimetic controls，孵育48 h后收集细胞。流式细胞仪法检测细胞的凋亡图（图 3A、图 3B）。图 3a表示姜黄素处理后与DMSO作为对照处理后细胞凋亡率。姜黄素处理后，细胞凋亡率为（8.6±0.9）%；DMSO作为对照处理后，细胞凋亡率为（3.0±0.4）%。与对照组相比，姜黄素处理后，细胞的凋亡率增加5.6%，姜黄素处理组与对照组比较有统计学意义（*n*=3, *P* < 0.05）；图 3b表示转染miR-186^*^ mimetics和mimetic controls后细胞凋亡率。转染miR-186^*^ mimetics后，细胞凋亡率为（1.5±0.4）%，转染miR-186^*^ mimetic controls后，细胞凋亡率为（3.1±1.0）%。与对照组相比，转染miR-186^*^ mimetics后，细胞凋亡率减少1.6%，miR-186^*^ mimetics与mimetic controls比较有统计学意义（*n*=3, *P* < 0.05）。细胞凋亡率=Q2或Q2-1象限早期凋亡细胞的比率。

**3:**
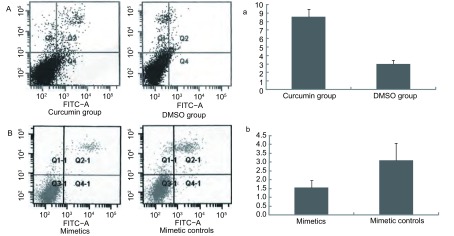
流式细胞仪法检测由miR-186^*^调控的细胞凋亡 The apoptosis of miR-186^*^ modulating detected by flow cytometry method

### 2.4 MTT法检测细胞存活率

15 μmol/L姜黄素处理和用0.02%DMSO作为对照处理A549/DDP细胞，24 h后，MTT法检测细胞存活率：以未处理细胞存活率为100%作参照，标准化后，得出姜黄素处理后，细胞存活率为（55.6±5.1）%。与对照组相比，姜黄素处理后，细胞存活率下降了44.4%，姜黄素处理组与其对照组比较有统计学差异（*n*=4, *P* < 0.05）；转染miR-186^*^ mimetics和mimetic controls，48 h后，MTT法检测细胞存活率：以转染miR-186^*^ mimetic controls后细胞存活率为100%作参照，标准化后，得出转染miR-186^*^ mimetics后，细胞存活率为（149.0±7.0）%。与对照组相比，转染miR-186^*^ mimetics后，细胞存活率增加了49.0%，miR-186^*^ mimetics与mimetic controls比较有统计学差异（*n*=4, *P* < 0.05）（图 4A、图 4B）。

**4:**
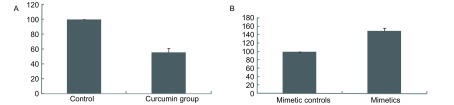
MTT法检测细胞存活率 The survival rates measured by MTT method

## 3 讨论

姜黄素是从姜黄、郁金、菖蒲等中药的根茎中提取的一种酚性化合物，具有降压、抗心肌缺血、抗凝、降脂等多种作用，已有文献^[10]^报道姜黄素对人胃癌细胞﹑结肠癌细胞等均具有明显抑制细胞生长和诱导肿瘤细胞凋亡的作用。耐顺铂人肺腺癌A549/DDP细胞株是由亲本人肺腺癌A549细胞株经DDP长期诱导形成的，已有文献^[11]^报道姜黄素对A549/DDP细胞的增殖有抑制作用。

目前体内外的研究清楚地表明异常表达的miRNA与癌症的发生有很大相关性。miRNA基因的表达仅占人类细胞有表达的所有基因的1%，但它被预测为可调控60%人类蛋白编码基因的表达^[12, 13]^，这就表明miRNA参与几乎所有基因通路的重要作用^[14]^。除此以外，大量证据表明：人类癌症的发生与miRNA的突变和异常表达密切相关，miRNA对人类癌症的病理学和生理学的研究起着举足轻重的作用^[15]^。掌握了这些知识，miRNA在肿瘤耐药细胞表达量的变化，将为临床对肿瘤的诊断和预后提供依据。换句话说，当研究聚焦在正常组织中miRNA与肿瘤耐药细胞中miRNA表达量比较时，在估计临床诊断和预后方面，更重要的是将miRNA的表达与肿瘤耐药细胞的表型或临床参数联系起来，从而为临床提供诊断和预后依据。

在肿瘤细胞中一些异常表达的miRNA可被清楚的分为致癌和抑癌基因。已有文献报道姜黄素通过调控胰腺癌细胞中的miRNAs的表达，从而产生对肿瘤细胞的抑制作用，并能诱导肿瘤细胞的凋亡。胰腺癌中miR-23a、miR-22、miR-21的表达比正常组织降低，姜黄素可以上调它们的表达^[19]^；反之，miR-7、miR-199a^*^、miR-92在胰腺癌细胞中的表达被上调，姜黄素可以下调它们的表达^[19]^，其参与的信号通路例如：姜黄素通过上调miR-22的表达并下调其下游靶基因*ESR1*的表达而抑制胰腺癌细胞的增殖^[19]^。以上说明姜黄素可能通过调控miRNAs的表达而促使肿瘤细胞凋亡。而且，大量的实验证据表明了姜黄素在肿瘤相关信号分子方面的可能的多种药理作用的复杂机理^[16-19]^。但姜黄素通过调控miR-186^*^的表达而诱导A549/DDP细胞的凋亡的研究尚未见报道。

miR-186^*^是一个新发现的分子，位于染色体1p31.1，我们通过microarray分析技术发现其表达水平在姜黄素处理的A549/DDP细胞中被明显下调，实时定量PCR验证了microarray分析结果。流式细胞仪法分别检测姜黄素处理后与DMSO作为对照处理后，A549/DDP细胞凋亡情况：与对照组相比，姜黄素处理后，细胞的凋亡增加5.6%；转染miR-186^*^ mimetics与mimetic controls后，与对照组相比，细胞的凋亡减少1.6%。MTT法检测姜黄素处理后与DMSO作为对照处理后，细胞存活率情况：与对照组相比，姜黄素处理后，细胞存活率下降了44.4%；转染miR-186^*^ mimetics与mimetic controls后，与对照组相比，细胞存活率增加了49.0%。由此我们的得出姜黄素抑制肿瘤细胞增殖的机理如下：姜黄素通过下调miR-186^*^的表达而抑制肺腺癌细胞的增殖。

综上，姜黄素抑制A549/DDP细胞的增殖和诱导细胞的凋亡可能是通过调控miR-186^*^而发挥作用的，然而所参与的信号通路还有待进一步研究。
